# Pennsylvania Hospital—Surgical Wards—Service of Dr. Peace

**Published:** 1850-12

**Authors:** 


### *Pennsylvania Hospital—Surgical Wards—Service of* Dr. Peace. *Cases discharged from March 1st to April 1st*, 1850.

| Disease. | Cured. | Average time under treatment in days. | By request. | Average time under treatment in days. | Died. | Average time under treatment in days. | Tot. |
|---|---|---|---|---|---|---|---|
| Abscess - - | 2 | 34 | 0 | | 0 | 0 | 2 |
| Burns - - | 1 | 69 | 0 | | 0 | 0 | 1 |
| Carbuncle - | 1 | 79 | 0 | | 0 | 0 | 1 |
| Caries - - | 0 | 0 | 2 | 19.5 | 0 | 0 | 2 |
| Contusion, - - | 4 | 28.5 | 0 | 0 | 0 | 0 | 4 |
| Corneal opacity | 0 | | 1 | 33 | 0 | 0 | 1 |
| Exostosis, - | 0 | | 1 | 273 | 0 | 0 | 1 |
| Fractures, 12 simple 7, viz.: | | | | | | | |
| Clavicle - | 0 | | 1 | 17 | 0 | 0 | 1 |
| Olecran. pr. | 1 | 31 | 0 | 0 | 0 | 0 | 1 |
| Radius - - | 1 | 41 | 0 | 0 | 0 | 0 | 1 |
| Metacarpus - | 1 | 34 | 0 | 0 | 0 | 0 | 1 |
| Leg - - | 3 | 53 | 0 | 0 | 0 | 0 | 3 |
| Compound, 5, viz.: | | | | | | | |
| Fingers - - | 1 | 50 | 0 | 0 | 0 | 0 | 1 |
| Thigh - - | 0 | 0 | 0 | 0 | 1* | 6 | 1 |
| Leg - - | 2 | 103 | 0 | 0 | 1† | 126 | 3 |
| Gonorrhœa - | 4 | 40.2 | 1 | 17 | 0 | 0 | 5 |
| Hæmorrhois - | 1 | 26 | 0 | 0 | 0 | 0 | 1 |
| Hare lip - - | 1 | 24 | 0 | 0 | 0 | 0 | 1 |
| Iritis - - | 1 | 21 | 0 | 0 | 0 | 0 | 1 |
| Inflammation of foot | 1 | 56 | 0 | 0 | 0 | 0 | 1 |
| leg | 1 | 24 | 0 | 0 | 0 | 0 | 1 |
| Sub. luxation - | 2 | 18.5 | 0 | 0 | 0 | 0 | 2 |
| Syphilis - - | 3 | 81.7 | 2 | 7 | 0 | 0 | 5 |
| Tumor - - | 1 | 39 | 0 | 0 | 0 | 0 | 1 |
| Ulcer - - | 1 | 35 | 1 | 102 | 0 | 0 | 2 |
| Wounds 9, viz.: | | | | | | | |
| Gunshot, - | 1 | 24 | 0 | 0 | 0 | 0 | 1 |
| Incised - - | 2‡ | 23.5 | 1 | 4 | 0 | 0 | 3 |
| Lacerated, - | 4 | 39.2 | 0 | 0 | 0 | 0 | 4 |
| Punctured - | 1‖ | 24 | 0 | 0 | 0 | 0 | 1 |
| | 41 | | 10 | | 2 | | 53 |

* Died of Tetanus. The patient fell from the top of a four storied house whilst running backwards upon the roof.

† This was complicated with simple fracture of the thigh in a depraved constitution.

‡ One of the larynx. ‖ Of the thorax.

